# Physiotherapists' Experiences Using the Ekso Bionic Exoskeleton with Patients in a Neurological Rehabilitation Hospital: A Qualitative Study

**DOI:** 10.1155/2020/2939573

**Published:** 2020-01-08

**Authors:** Emily Read, Cora Woolsey, Chris A. McGibbon, Colleen O'Connell

**Affiliations:** ^1^Faculty of Nursing, University of New Brunswick, Canada; ^2^Institute of Biomedical Engineering, University of New Brunswick, Canada; ^3^Faculty of Kinesiology, University of New Brunswick, Canada; ^4^Faculty of Medicine, Dalhousie University, Canada

## Abstract

Use of bionic overground exoskeletons to assist with neurological rehabilitation is becoming increasingly prevalent and has important implications for physiotherapists and their patients. Yet, there is a paucity of research about the impact of integrating this technology on physiotherapists' work. The purpose of this study was to explore how the training and implementation of using the Ekso robotic exoskeleton with patients affects physiotherapists' work. An exploratory qualitative study of three physiotherapists working at a neurological rehabilitation centre in Eastern Canada was conducted using one-on-one semistructured interviews in July 2017. Audio recordings were transcribed verbatim, and data was coded and analyzed using thematic analysis. Six themes emerged from the data: developing organizational capacity; ethical use of technology; benefits of the equipment; challenges of the equipment; cognitive workload; and the technological environment. The results suggest that the adoption and integration of bionic exoskeletons into rehabilitation practice is not as simple as training physiotherapists and giving them the device. More research is needed to understand the increased cognitive demands of working with patients using technologically advanced exoskeletons within a dynamic, technology-rich healthcare environment, while managing patient expectations and ethical use.

## 1. Introduction

Impaired mobility is a major concern in rehabilitation and can be caused by arthritis, stroke, neural injury from traumatic brain injuries or Parkinson's disease, and spinal cord injuries (SCIs) [1, 2]. SCIs are prevalent globally, are often devastating to individuals and their families, and are expensive to treat and rehabilitate [3]. Worldwide, 24 million individuals were living with SCIs in 2016 [4]. Stroke-related paralysis is the most common adult physical impairment, requiring intensive rehabilitation to recover functionality [5]. Mobility impairment can cause secondary degenerative changes in muscles and neural processes [6] and can lead to physical complications such as muscle atrophy and pain [7] as well as psychological and emotional issues such as depression [8]. Loss of mobility can also have catastrophic impacts on patients' work lives, relationships, and community participation [5]. Solutions to improve patients' quality of life, ease the strain on the healthcare system and caregivers, and promote functional independence and vocational/community reintegration are therefore of critical importance. Such needs will only increase [9], as the global burden of disease shifts from communicable disease to persons living with sequelae of chronic disease and physical trauma [10].

Improving lower extremity function and, if possible, recovering the ability to walk are important priorities for individuals who have lost locomotor function and are typically a focus of rehabilitation therapies [6, 7]. Assistive devices such as walkers, reciprocating gait orthosis, and wheelchairs augment individuals' mobility and can aid in rehabilitation of muscle and neural processing, but they do not necessarily allow individuals to access the whole range of daily activities or sufficiently train individuals to regain lost motor skills [11]. Limitations in locomotion mean that basic ambulation such as balanced standing or taking a few steps can cause fatigue, even with the physical support of a physiotherapist or walking aid [2]. Additionally, traditional rehabilitation methods often necessitate body weight support for the patient, which is physically exhausting for physiotherapists [12, 13] and may cause job-related pain or injury [14].

In recent years, robotic exoskeletons have emerged as a gait rehabilitation option that can alleviate some of the physical demands of lower extremity rehabilitation. There are currently several different rehabilitation exoskeletons available from different companies [15]. While not identical, their general design consists of an external motorized structure fitted over and around weak or paralyzed lower extremities to help with standing, walking, and other activities of daily living [6]. Exoskeletons are controlled by either the physiotherapist or the wearer using a control panel or trigger or prompted by the wearer with their leg or upper body movements, such as leaning forward.

Exoskeleton-aided locomotion has several advantages over traditional gait training and rehabilitation, including lower exhaustion rates for patients and physiotherapists [12], improved gait motion [16], and expanded terrain and location options for training [6]. Most, but not all, exoskeletons fully support the body weight of the wearer, allowing patients to walk fully upright using proper body mechanics, which potentially strengthens muscles and neural motor skills that would not otherwise be engaged [11, 17]. Compared with conventional gait training, robotic exoskeletons (like Ekso) have been shown to greatly increase the amount of work that can be done by low- or nonfunctioning lower limbs, representing a revolution in rehabilitation [18].

The Ekso is used by several rehabilitation clinics around the world and has been evaluated in studies that looked at biomechanics and patient safety [8, 11]. Research has demonstrated many physical benefits for patients using the Ekso device including better balance, decreased spasticity, stronger core muscles, improved mental health, better bowel control, and improved gait [15, 19–23]. Kolakowsky-Hayner et al. [7] showed that the Ekso device was safe to use with patients with SCIs and did not cause pain, fractures, swelling, or skin degradation. Høyer et al. [23] found that 8–9 gait training sessions using Ekso resulted in significant increases in walking time and number of steps and improved strength and postural control for patients with hemiplegia poststroke (see also [24]). Patients also reported high satisfaction with Ekso sessions [11]. Kozlowski et al. [25] found that the learning trajectory varied based on patient characteristics and most required some assistance to use the device. The authors also noted anecdotally that some patients reported more regular bowel movements, improved balance and posture while sitting, better sleep, and decreased pain and spasticity and that all patients wanted to keep walking in the Ekso regularly. Brenner [20] found that the psychosocial impacts of using the Ekso to rehabilitate veterans in Denver, Colorado, included improved mood, reduced stress levels, and increased self-satisfaction. Ekso use has also been shown to improve physical performance outside of therapy sessions, including increased time spent walking, greater control over posture, weight shift, and mobility and reduced use of nontherapy aids like walking canes [23]. Overall, initial findings suggest that there are important physiological and psychosocial implications of using the Ekso for gait training and rehabilitation that need further exploration.

As described, the majority of studies on robotic exoskeletons in physiotherapy to date have focused on biomechanics, safety, and clinical outcomes. Few studies have examined the perspectives of patients, and even fewer have investigated physiotherapists' perspectives on exoskeletons, despite the central and essential role of physiotherapists in rehabilitating patients with gait impairment. No published studies, to our knowledge, have explicitly looked at rehabilitation with exoskeletons from an operational standpoint. The impact of robotic exoskeleton training and use on the worklife and practice of physiotherapists has been largely unexplored. In our review of the literature, we found one study [7] that reported the total average Ekso set-up time for practitioners including device preparation, participant transfers, donning, and doffing (18.13 minutes). However, the study did not explore the experience or implications for physiotherapists' practice using the Ekso. The current study examined how physiotherapists at a rehabilitation centre in Eastern Canada view their work with overground ambulatory training using the Ekso GT exoskeleton as well as their perceptions of the impact its use has had on their patients.

## 2. Methods

### 2.1. Study Design

Due to the novelty of this research and the small number of physiotherapists using the Ekso, an exploratory qualitative research design using a constructivist thematic analysis methodology [26] was employed, which allowed us to gain a rich understanding of the participants' experiences, using their own words and perspectives.

### 2.2. Setting

The study took place at a neurological rehabilitation centre in Eastern Canada. This facility has inpatient and outpatient clinical and research programs for adults and children with neurological conditions.

### 2.3. Participants

Three full-time physiotherapists who worked with adult patients (age 18 and above) in the inpatient and outpatient units of the centre were invited and agreed to participate in the research study. All participants had undergone Level 1 and Level 2 training with the Ekso in the Spring and Summer of 2017 and used it with patients as part of their practice-based research. These were the only physiotherapists using the Ekso at this facility which limited the number of possible participants for the study.

### 2.4. Description of the Technology

In this study, participants were using the Ekso GT, a robotic exoskeleton made by Ekso Bionics and approved for use with individuals with SCIs and hemiplegia due to stroke [27]. The Ekso device is a battery-powered suit made of carbon fiber and uses a hydraulic power system [11, 28]. It attaches to the individual at their torso by a backpack, at the thigh, calf, and foot of each leg, and has motors at the hips and knees to enable the user to stand and step overground with weight-bearing and gait [11, 28]. Sensors are embedded into the braces that fit around the user's body, which respond to muscular output from the user [11]. It is the fastest exoskeleton currently available with a top speed of 0.89 m/s [11]. The Ekso operates in a number of modes depending on the level of assistance required for the patient and also trains transition movements such as sitting to standing and standing to sitting [12].

### 2.5. Data Collection and Analysis

Research ethics approval for the study was obtained from the regional health authority of the neurological rehabilitation centre and the researchers' university. The three clinician-research physiotherapists who had been trained and worked with the Ekso at the facility were personally invited by the lead researcher to participate in a one-on-one interview at a mutually agreeable time. Before starting each interview, participants were given a letter of information about the study and provided written consent to participate. All interviews were recorded with a digital audio recorder and transcribed verbatim. Transcripts were then analyzed for significant themes.

Two researchers independently assigned codes to the texts of transcripts and then met to discuss overlap and differences and to organize codes into overarching themes. Analysis initially focused on understanding the data, followed by assigning codes and developing overarching themes. Inductively constructed codes were used to categorize statements that shared similar ideas including topics of discussion in response to questions and areas of agreement and disagreement among participants. During discussion, codes that did not fit into any larger themes (i.e., were not related to any other codes) were either assigned to their own theme or discarded depending on whether the researchers identified them as integral to the experiences of physiotherapists. Where the researchers disagreed, codes were discussed until consensus was reached. The researchers then independently went through the texts again and verified the themes to determine how well the participants' words fit the themes. The researchers met again to discuss the themes and to draw out evidence for each theme (e.g., quotes, latent meanings, and confirmation from the literature). If themes confirmed evidence from the literature, the researchers framed their discussion of the themes in terms used by researchers who had previously identified the phenomena.

## 3. Results

Six themes emerged from the data: developing organizational capacity; ethical use of technology; benefits of the equipment; challenges of the equipment; cognitive workload; and the technological environment.

### 3.1. Developing Organizational Capacity

The process of developing greater organizational capacity by working with the Ekso emerged as an important theme. All three respondents spoke of how the Ekso has enhanced their practice, increasing what can be done within the context of neurorehabilitation therapy. These gains include specialized expertise in a cutting-edge rehabilitation technique and piece of technology, greater physical capacity of the therapists to work with clients (fewer physiotherapists needed, particularly with inpatients, and greater ability to physically support patients), and an increase in what patients can accomplish in their sessions.

The results also suggested that the Ekso strengthens rehabilitation physiotherapy practice in several ways. First, the requirement for a team of two or more physiotherapists and physiotherapist assistants builds a sense of teamwork. For instance, one participant noted that

[During] the initial session, we always are still sticking to the three of us working through it together just to kind of have another perspective … somebody else to talk things over with.

This provides the opportunity to collaboratively develop protocols and discuss the appropriateness of the Ekso for each patient and also continuously builds expertise in the use of the device. The same participant also emphasized that practice and repetition were important to developing and maintaining their expertise:

That's one good thing about … not having a lot of us trained, I am pulled in a lot to do it which is repetition, what I need to maintain my skills.

Second, the Ekso increased the physical work capacity of patients and physiotherapists alike. All three respondents noted that the Ekso allows patients to walk further than with conventional overground training because both the physiotherapist and the patient do not become exhausted as quickly. For example, one participant noted that

Repetition and mass practice are the biggest things we can do so if you have somebody who's low-functioning and is just starting to get on their feet, as a PT it's a lot of work to do that gait training with them. We fatigue, they fatigue, and then you might get ten steps in a session, so what we're really enjoying with the Ekso is with that same client I can put them in [the Ekso] and I can do 400 steps, so that's huge, for motor planning and for repetition.

The same participant also noted that the Ekso is safe for patients, allowing them to take breaks while in the device without having to stop the entire gait training session.

Overall, the significant investment of time and resources required to use the Ekso was recognized and perceived to be worthwhile considering the return on investment for their patients, themselves, and their organization.

### 3.2. Ethical Use of Technology

Another theme that emerged from the interview data was the importance of ethical use of the Ekso technology. Participants reported that the Ekso has stimulated conversations centred on developing an ethical practice and managing the expectations of patients who would be using it. The physiotherapists conceptualized using the Ekso in their practice is a privilege, and they felt a strong sense of accountability regarding its use. This sense of privilege stems not only from the cost of the equipment but also from the major advance the Ekso represents for patient outcomes, the awareness that it is not available at most clinics and to most patients, and the opportunities that the physiotherapists have to be engaged in cutting-edge clinical research. The participants all indicated that developing ethically sound practices around the Ekso is built into their use of the technology and their practice as physiotherapists. Accountability includes giving students the opportunity to work with the Ekso, managing the patient-Ekso relationship to ensure appropriate use and expectations, and working out general guidelines for what constitutes appropriate patient goals for using the technology.

The Ekso was purchased for both research and eventual integration in rehabilitation practice, so the participants felt that they need to be cognizant of multiple goals and considerations when they recommend the Ekso to a patient. The participants each talked about managing patient expectations. One participant remarked that

people hear that we have it and they see it and … obviously people want to try it ‘cause it's technology and it's cool.

However, the participants felt that the use of the Ekso needed to be justified. Justifications for use were based on criteria developed collaboratively by the physiotherapists drawing on their expertise and experience and were continuously being updated and revisited in group discussions. These conversations appear to be an important source of peer support for using the Ekso as they provide guidelines, flexibility, and collegial feedback.

Our results suggest that the time it takes to develop this sense of ethics and code of accountability is substantial and involves significant collaboration with colleagues in an ongoing process. The sense of privilege seems to stem from the large degree of benefit that the respondents feel they and their patients receive from Ekso. Two respondents felt “fortunate” to have access to the Ekso, and one felt that their patients were very aware that the technology is not widely available and were “thankful.” This sense of gratitude to have access to the Ekso seems to be part of a larger feeling of accountability common to all three participants and a desire to give the best care possible to their patients.

### 3.3. Benefits of the Equipment

Respondents were all very clear that they felt the benefits of the Ekso were numerous and worth the investment of time and resources. Patients experienced the greatest benefits, which were severalfold. For example, patients could take many more steps with the Ekso than without, allowing much more opportunity to work on balance, gait, and core strengthening than would otherwise be possible. One participant felt that the Ekso gave patients the opportunity to concentrate on stepping instead of balance:

The Ekso gives them more balance so they're not focusing so much on their balance. They can focus more on the gait aid … instead of having to put so much focus into where their feet are that they can't even think about moving the walker ahead or moving the cane.

Patients also are able to remain upright longer before tiring and to rest in the Ekso suit safely. In addition to improving the quality and quantity of physical therapy, it was reported that some patients experienced physical gains in their activities of daily living as a result of using the Ekso.

The physiotherapists highlighted that there were psychological and emotional benefits for nonambulatory patients because Ekso allows them to walk upright; this causes patients to look forward to their sessions. One participant spoke of a patient whose emotional wellbeing was improved by using the Ekso:

As soon as you would put him up in the Ekso his face would just light up. He would just be so happy.

The participant also noted that

[Patients are often] very proud at the end to see that they were able to do that 400 steps or whatever we did in that session; it really plays a big key role in their emotions.

To a lesser extent, participants cited benefits to themselves. These included the ability to support patients using fewer people and the slower fatigue rate resulting from having to use less physical strength to support patients during gait training. This was especially true when working with inpatient clients. Another benefit was recognizing the possibility of doing research using the Ekso. All three participants acknowledged that the Ekso is more beneficial for some patients than for others; it is particularly useful in cases where patients have recent injuries, whereas patients who have lived with immobility for longer periods are less likely to respond well. However, one participant felt that, even in the case of a patient who would never likely walk again, the Ekso strengthened core muscles not possible to work using a wheelchair, and the patient's wheelchair tennis game improved significantly as a result.

### 3.4. Challenges of the Equipment

Respondents overwhelmingly cited the investment of time required to learn to use the device as the largest challenge of using the Ekso. One participant emphasized that the Ekso is “therapist-intensive, in terms of the training and the knowledge you have to have.” The training and certification to use the Ekso is more in-depth than other kinds of technology, and learning to use it continues after the training is over. While one respondent viewed the Ekso as “just another tool,” there is no doubt that managing the many dimensions of using the technology (skill acquisition and maintenance, management of patient expectations, physical demands, and assessment of patient needs) contributes to the cognitive workload of the physiotherapists using it.

Moreover, the Ekso requires a large block of time for a single session (60 min) and a larger block for the initial assessment (90–120 min), so time constraints on patients' schedules sometimes cause difficulties. The highly technical aspect of the Ekso means that physiotherapists must dedicate a large amount of time to properly fit and adjust the device to the patient during assessments and in each session, in addition to the large amount of time required to be properly trained in the use of the Ekso. One participant noted that

Initially we were all maybe a bit overwhelmed by the amount of information and how different it was from anything that we have used in the past.

The participant went on to note that

The biggest time consumption is during the assessment phase.

Another challenge is that it can cause anxiety in some patients because it is a different mode of movement than they are used to and requires the patient to give up some motor control. Additionally, a poorly fitted Ekso can cause discomfort, as can a larger degree of patient spasticity—as, for instance, can be triggered when a patient is feeling anxious about the equipment. This means that physiotherapists need to acquire the skills to properly fit the device to each patient and to constantly monitor to make sure the patient can handle being in the device during each session. Also, the Ekso does not fit everyone, so only certain patients can use the technology. This means that physiotherapists need to manage patient expectations around whether the Ekso is appropriate.

One important issue that emerged in the interviews is that the Ekso, and other technologies like it, cannot completely take the place of conventional therapies like stretching. Therapists must choose which strategy to pursue given the limited time they have with each patient. Because of this, they need to be clear about their rationale for using the Ekso as opposed to other therapies. All of the participants worked several patients using the Ekso but with many patients who did not use the Ekso, either because they did not fit the criteria or because other therapies were considered more appropriate. Our results suggest that developing criteria and protocols through discussion is the main way the participants deal with this issue.

### 3.5. Cognitive Workload

The participants each made reference to managing a complicated set of physical, ethical, and logistical factors in using the Ekso with patients. Although participants did not explicitly articulate any concept such as workload or burden, all three did describe an intimidating set of considerations in using it with patients. These include the requirement for Ekso-specific technical know-how, a concern with maintaining patient safety, the necessity of working within time constraints, a set of criteria for the appropriateness of the technology in a given situation, an ability to accurately measure the numerous measurements required for proper fit, a need to check and recheck that all is operating as it should, and an understanding of patient needs, all in addition to traditional PT knowledge, skill, and mental workload. They described the training sessions and the subsequent therapy sessions as an unspecified workload of “a lot,” “a lot of information,” “tiring,” or “challenging,” indicating that a great deal of multifaceted mental work was put into functioning as an Ekso expert and physiotherapist. As mentioned earlier, one participant also felt that learning to use the Ekso was “overwhelming,” and all three participants felt that maintaining the skills required for using the Ekso needs to be constantly maintained or they would have to reread the manuals. The requirement for skill maintenance and the physiotherapists' feeling that they are always learning about the Ekso suggests that operating the Ekso with a client requires a high and sustained level of cognitive workload.

### 3.6. Technological Environment

The physiotherapists reported that they work within a technologically dense work environment into which the Ekso has been introduced. Ekso is somewhat like the other technologies in that it is a tool to be used in physiotherapy, but unlike other tools, it is more learning- and time-intensive. This technologically dense work environment is an important part of understanding the cognitive workload of physiotherapists. The Ekso was perceived as more or less intensive by physiotherapists depending on their technological skill, comfort level, and constellation of knowledge. One participant, comparing the skill levels of physiotherapists in using the Ekso, noted that some physiotherapists are more comfortable with controlling robotics, possibly due to their age and/or interests, such as playing video games. The fact that only some therapists have the opportunity to use and develop skills around technology appears to create two tiers of physiotherapy, and patients may seek out physiotherapy with technological options if it is available to them. This requires that physiotherapists manage patient expectations of the Ekso, which may not be appropriate for all patients.

## 4. Discussion

The results of our thematic analysis point to physiotherapists needing space and time to develop an ethical practice and clear protocols around using the Ekso. All three participants referred to discussions and collaboration that constitute a deliberate reflexive practice. Since using the Ekso is cognitively intensive and requires both formal training and practice-based learning, protocols can help lower the amount of mental workload required to use the device. The participants all referred to an ongoing effort to work out ethical considerations, sound but flexible criteria for using the Ekso, and strategies for managing patient expectations as part of an overall feeling of accountability. Our findings suggest that use of the Ekso requires significant time for physiotherapists to reflect on their practice and build discursive knowledge through collaboration and discussion. This requirement for organizational support (e.g., time for discussions) is consistent with the findings of Glegg et al. [29] who found that physiotherapists' adoption of technology is dependent to some extent on the willingness of their organization to support them with additional resources including time for program development and troubleshooting.

The participants' experiences confirmed many of the findings of other researchers about the benefits of the Ekso in gait training. In agreement with Androwis and Nolan [19] and others [6, 15], the participants noted improvements in gait, core strength, bowel control, balance, and mental health, and like Kolakowsky-Hayner [7], they found that the Ekso is safe. They also observed that the Ekso allows significantly more work to be done during a session, in agreement with much of the literature on robotic exoskeletons. The participants also cited benefits beyond biomechanical factors that have not been explored in the literature. The Ekso provides the participants with the opportunity to build teamwork and collaborative practices, to strengthen ethical practice, to introduce students to new technologies, and to provide extra motivation to patients with the “cool” factor [30]. The Ekso therefore appears to build capacity in organizations in ways that are harder to define yet nonetheless contribute to the strength of the practice.

The findings highlighted the importance of considering the technological environment and physiotherapists' cognitive workloads when integrating the Ekso into an existing practice. Since exoskeletons are relatively new, many studies have focused on lab settings, smaller sample sizes, and limited outcome measures. Meta-analyses of these studies show that, though the technology is promising, insufficient data exists about the contexts in which exoskeletons achieve the best results [11]. The role physiotherapists play in the integration of technology and new practices is crucial, so providing physiotherapists with adequate training, time, and resources is likely a key factor in successful integration. Studies of physiotherapists' workload have shown that this sector is vulnerable to work-related pain and injury, burnout, and workplace stress [14, 31, 32]. Physiotherapists usually operate within a technologically dense environment, so introducing new technology may not present the same difficulties for frontline workers as has been documented in other healthcare situations [33]. However, technology is often introduced into healthcare organizations without evaluating the impact on the healthcare workforce and patients holistically [33]. As a result, technology can ultimately act as a barrier between management and health practitioners [34], violate patients' rights, or increase workplace stress and burnout (Selmanovic 2011). Additionally, physiotherapists are sometimes subjected to top-down management that does not take into consideration best practices and may result in workplace stress and burnout [32]. Studies of barriers to technology implementation often do not consider the needs of frontline workers in changing technological and operational environments, leading to negative outcomes, such as workers' dislike for technology and disloyalty to an organization [33].

These organizational and human factors need to be considered in integrating the Ekso into an existing practice. Since the Ekso is cognitively intensive and requires significant investment during training and subsequently with patients, poor management of Ekso integration could result in poor patient outcomes and increased burden on physiotherapists. Rehabilitation practices are typically technology-rich work environments, requiring a high-level baseline of technological literacy and comfort from physiotherapists. Our findings highlighted the significant increase in technological know-how required of physiotherapists using the Ekso.

### 4.1. Limitations

One limitation of our study is the small sample size. While the Ekso is being used at an increasing number of rehabilitation facilities worldwide, it is still a highly specialized and novel piece of technology that requires a significant investment. For this reason, there was only one Ekso and three physiotherapists trained to use it at our research site. In our experience, this is consistent with the number of devices and trained staff at other facilities. However, despite the small sample size, the findings of our pilot work demonstrate that physiotherapists' experiences working with this technology are important to understand and consider when contemplating or planning its adoption into clinical rehabilitation practice. Future research using a multisite study design to increase the number of participants is recommended to increase the robustness of the findings and compare experiences across sites and internationally. We also feel that a cognitive task analysis of physiotherapists using the Ekso would uncover significantly more about the challenges and benefits of using the Ekso. Additionally, a study of the cognitive demands on patients using the Ekso would give further insights into successful integration into clinical practice.

## 5. Conclusion

To our knowledge, this is the first study exploring the use of the Ekso from the perspective of physiotherapists. Our findings were consistent with past literature on exoskeletons concerning the benefits over conventional gait training such as the large increase in work that can be accomplished by a patient within a session, better balance and gait, improved bowel control, and improved mental health. Novel findings were that participants also discussed the collaborative aspect of using the Ekso and the space opened up for developing ethical practice and protocols, aspects that have not been explored in the literature on exoskeletons. The importance of group discussions and protocol development appears to lie in lowering the cognitive workload, such that PTs are able to follow protocols that have been collaboratively developed, seek feedback from colleagues, and revise protocols if they feel they have justification for doing so. Moreover, the findings suggest that organizational support and consideration of the technological environment and technology literacy are key factors to consider when adopting and integrating an exoskeleton into physiotherapists' practice.

## Data Availability

We do not currently have REB approval to share the interview transcripts with others outside of the research team.
